# Dasatinib associated lymphadenopathy in a chronic myeloid leukemia patient: A case report

**DOI:** 10.1097/MD.0000000000022791

**Published:** 2020-11-06

**Authors:** Dimitrios Pilalas, Triantafyllia Koletsa, Georgios Arsos, Grigorios Panselinas, Paraskevi Exadaktylou, George Polychronopoulos, Christos Savopoulos, Georgia D. Kaiafa

**Affiliations:** a First Propedeutic Department of Internal Medicine, AHEPA University Hospital, Medical School; b Pathology Department, Medical School; c 3rd Department of Nuclear Medicine, Papageorgiou Hospital, Medical School, Aristotle University of Thessaloniki, Greece.

**Keywords:** chronic myeloid leukemia, dasatinib, lymphadenopathy, case report

## Abstract

**Rationale:**

Dasatinib associated lymphadenopathy (DAL) is a rare adverse event in chronic myeloid leukemia patients (CML). A case of voluminous lymphadenopathy in the context of DAL is presented.

**Patient concerns:**

A 40-year-old male patient was diagnosed with BCR-ABL1 positive chronic stage CML 2 years ago and achieved complete molecular response on nilotinib, which was switched to dasatinib due to nilotinib intolerance. After 5 months on dasatinib, the patient presented with a large mass in the axillary region.

**Diagnosis:**

Common infectious and autoimmune etiologies of lymphadenopathy were ruled out. The positron emission tomography/computed tomography (PET/CT) demonstrated a hypermetabolic lymphadenopathy highly suspicious of lymphoma. The subsequent biopsy excluded lymphoma or extramedullary blastic transformation of CML and revealed reactive lymphadenopathy with mixed (cortical and paracortical) pattern. Clinical history and clinicopathological correlation suggested the diagnosis of DAL.

**Intervention:**

Dasatinib was discontinued and the patient remained in close follow-up. TKI treatment with nilotinib was reinitiated.

**Outcomes:**

Lymphadenopathy resolved clinically at 4 weeks and normalization of PET/CT findings was documented at 9 weeks after cessation of the drug. TKI treatment with nilotinib was reinitiated with good tolerance.

**Lessons:**

DAL may present with voluminous lymphadenopathy consistent with malignancy in clinical and imaging workup. We describe the spectrum of lesions associated with DAL and identify common features with drug-induced lymphadenopathy.

## 1 Introduction

Tyrosine kinase inhibitors (TKIs) have revolutionized chronic myeloid leukemia (CML) treatment rendering the patient life expectancy comparable to life expectancy of the general population through lifelong daily administration of a generally well-tolerated oral medication.^[1]^

The safety profile varies among different agents and frequently the clinician must balance between an effective dose of a particular agent and the associated adverse events. Common adverse events include nausea/vomiting, diarrhea, muscle cramps, edema and cytopenias; less commonly severe vascular, cardiac, pulmonary, metabolic/endocrine and hepatic toxicities may arise.^[1]^ Some of these adverse events could be attributed to the limited specificity of the currently available TKIs resulting in the inhibition of other kinases, but further study is necessary to elucidate the underlying mechanisms.^[2]^

In rare instances, CML patients on dasatinib treatment may experience lymph node enlargement reversible upon dasatinib discontinuation.^[3]^ Herein, we present an analogous case with focus on the clinicopathological and imaging features as well as the associated differential diagnostic problems.

## 2 Case report

We present a 40-year-old male CML patient on dasatinib who developed lymphadenopathy. The patient was diagnosed with BCR-ABL1 positive chronic stage CML (low risk group ELTS score) 2 years ago following a workup for fatigue and left upper quadrant abdominal discomfort. Although treatment with nilotinib was effective with documentation of complete hematologic remission at 3 months and complete molecular response (MR^4,5^) at 1 year, the tolerance was suboptimal with recurrent episodes of maculopapular rash eruption and periorbital edema exacerbating the pre-existing allergic conjunctivitis of the patient. CML treatment was switched to dasatinib and 5 months later the patient consulted for evaluation of a non-tender left axillary mass of recent onset.

Except for the voluminous lymphadenopathy, the patient was asymptomatic, and physical examination was notable for a maculopapular rash at resolution. No relevant epidemiologic risk factors were identified. A complete blood count was normal, serologic testing for Epstein-Barr virus, cytomegalovirus, herpes simplex virus-1 and varicella-zoster virus showed no evidence of recent infection and testing for human immunodeficiency virus, toxoplasmosis and herpes simplex virus-2 was negative. Antinuclear antibodies and a tuberculin skin test were negative as well.

**Figure 1:**
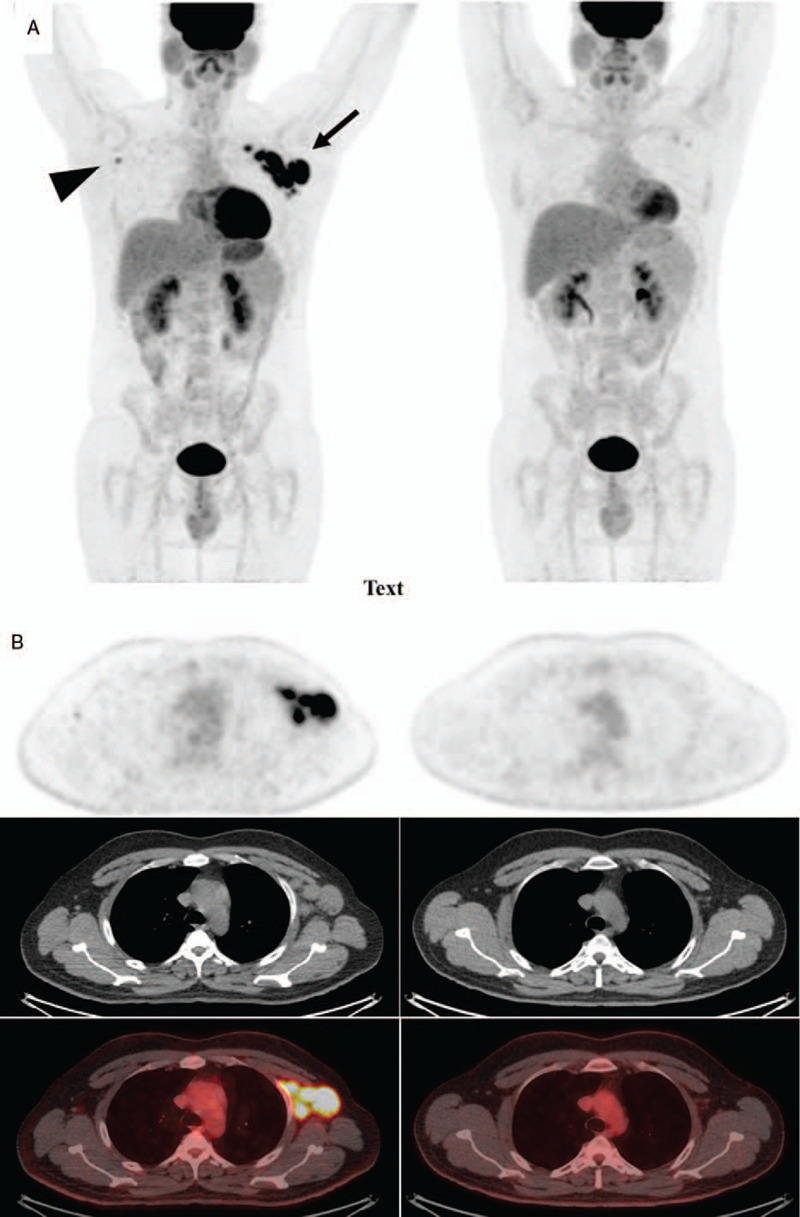
^18^F-FDG PET/CT scans on (left) and 2 months off dasatinib (right). A) Maximum intensity projection images. Left, intense tracer uptake (SUVmax 16.7) is observed in grossly enlarged left axillar and subclavian lymph nodes (arrow) and moderate (SUVmax 4.2) in a small right axillar lymph node (arrowhead). Right, almost complete metabolic normalization of the left axillar lymph nodes with only mild metabolic activity seen in a pair of subclavian lymph nodes and complete normalization of the right axillar lymph node. B) Axial sections at the level of axilla. Upper row, PET sections; middle row, CT sections; lower row, PET/CT sections.

The patient underwent a ^18^F-fluoro-deoxyglucose (^18^F-FDG) positron emission tomography/computed tomography (PET/CT) scan with above head to mid-thigh images acquired using a GE Discovery 710 TOF PET/CT scanner 60 minutes after the intravenous tracer administration; attenuation corrected PET images were reconstructed in the 3 main anatomical planes and fused with the corresponding CT sections. Highly increased tracer uptake (SUV_max_ 16.7) was documented in grossly enlarged left axillar and subclavian lymph nodes and moderate (SUV_max_ 4.2) in a small right axillar lymph node (Fig. 1A).

**Figure 2:**
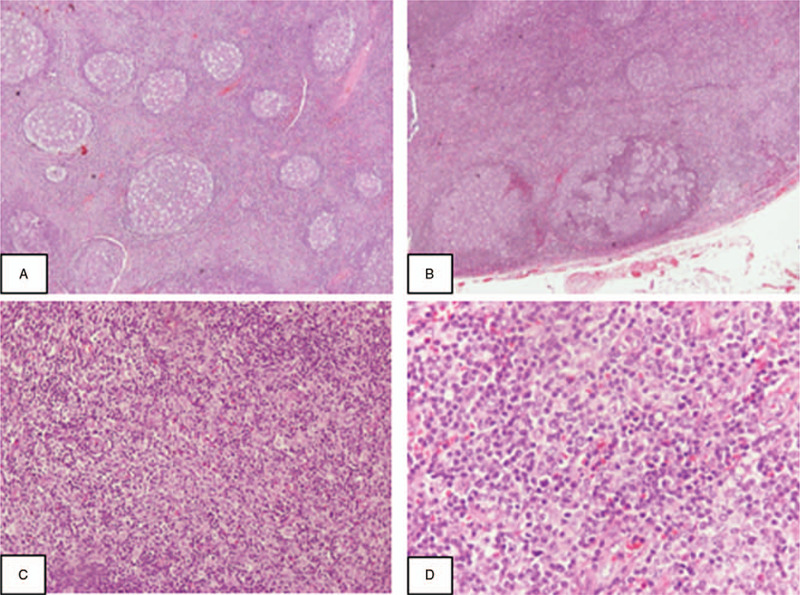
Morphological features of lymph nodes in dasatinib-associated lymphadenopathy: A) Preservation of the architecture with follicular hyperplasia. B) Progressive transformed germinal centers. C) Paracortical hyperplasia. D) immunoblastic proliferation (A,B: HE X40; C: HE X200; D: HEX400).

A block of left axillary lymph nodes was excised and sent for histologic examination. The specimen measured 5.5x5.5x2.6 cm and was fixed in formalin buffer. Hematoxylin and eosin stained sections revealed preservation of the nodal architecture with follicular hyperplasia (Fig. 2A). Follicles were of different size and maintained their normal polarity and tingible body macrophages in the germinal centers. There was also a small number of follicles with progressive transformation of germinal centers (PTGCs) characterized by mantle zone lymphocytes invading the germinal centers (Fig. 2B). Germinal center cells were CD10 and BCL6 positive and BCL2 negative. Immunostaining with CD21 and CD23 highlighted the follicular dendritic cell meshwork. In addition, areas presenting expansion of the paracortex of the lymph node were also observed. These areas were characterized by a polymorphous infiltrate with the presence of eosinophils, plasma cells and lymphocytes of variable size including large activated lymphocytes that resemble immunoblasts (Fig. 2C). Few of the activated lymphoid cells exhibited multilobular nuclei (Fig. 2D). These cells were positive mainly to B-cell (CD20, PAX5) markers. Only few of them were immunoreactive to T-cell markers (CD3, CD5) or CD30 antibody, whereas CD15 and EMA immunostains were negative. There was no rimming of the large cells by T- cells or CD57+ cells, which were few and scattered. Plasma cells showed polytypic expression for kappa and lambda light chains. In situ hybridization for Epstein-Barr virus (EBER) was negative. Based on these histological findings the diagnosis of reactive lymphadenopathy was set, with mixed (cortical and paracortical) pattern and with progressive transformation of the germinal centers.

The hypermetabolic findings of the PET/CT being highly suggestive of lymphoma prompted a meticulous examination of the whole specimen. However, the aforementioned diagnosis of reactive lymphadenopathy was retained with features compatible with drug induced lymphadenopathy. These findings have been previously described in patients treated with dasatinib or other drugs, specifically anticonvulsants; dasatinib was suspected as the etiological factor and discontinued. The patient was followed-up every 2 weeks for 2 months. The patient maintained hematologic remission and the lymphadenopathy subsided 2 weeks after dasatinib withdrawal and was clinically undetectable at 4 weeks. Nine weeks after treatment interruption a new PET/CT scan was performed documenting an almost complete metabolic normalization (Fig. 1B). Of note, administered ^18^F-FDG activity, body weight and blood glucose levels were comparable in the 2 instances (7.6 mCi, 76 kg, 99 mg/dl vs 8.0 mCi, 77 kg, 104 mg/dl). TKI treatment with nilotinib was reinitiated with good tolerance.

## 3 Discussion

The differential diagnosis in a CML patient with localized lymphadenopathy may present significant diagnostic challenges. Along with the typical workup, the exclusion of extramedullary blastic transformation of CML is warranted. Lymphoma has scarcely been described in the literature in CML patients,^[4]^ but patients on dasatinib treatment may present lymph node enlargement characterized by follicular hyperplasia, with or without PTGC morphology^.[4,6]^ To our knowledge, 17 cases of DAL have been described in the literature.^[5,6]^ The mechanism of the follicular hyperplasia remains unknown. It has been assumed that TKIs involved in B-cell activation through the BCR and the Akt pathways could promote B-cell proliferation.^[2]^ However, in some cases, including ours, additional paracortex expansion due to a heterogeneous population of eosinophils, plasma cells, histiocytes and immunoblasts, some of which with characteristics of lymphocyte predominant (LP) and Hodgkin and Reed/Sternberg (HRS) cells are present.^[6–8]^ Taken together, these histologic findings have been described in lymphadenopathy associated with anticonvulsant therapy, more commonly with phenytoin, as well as with other drugs, and represent a drug-induced lymphadenopathy. ^[9,10]^

The intense ^18^F-FDG uptake by enlarged lymph nodes being the metabolic imaging hallmark of high-grade lymphomas, heightened the index of suspicion of lymphoma in our case. However, this PET appearance is far from lymphoma-specific as any kind of fast proliferating or reactive cells, like in the context of inflammation, can also concentrate ^18^F-FDG avidly. Overexpression of the cell membrane bound glucose transporter (GLUT) molecules family, is an important first step of this process. In particular, reactive B-cells in PTGCs, have been shown to display strong GLUT1 expression.^[11]^ This phenomenon, along with reactive follicular hyperplasia, harboring a variety of hypermetabolic, dividing cells, can lead to false positive ^18^F-FDG PET/CT results in both non-malignant and lymphoma conditions.^[12]^ Among dasatinib associated lymphadenopathy cases, 2 reported PET/CT results with hypermetabolic lymphadenopathy but further details were not provided.^[3,5]^ It should be noted that hypermetabolic lymphadenopathy in PET scan has been described in drug induced hypersensitivity syndrome (DIHS) secondary to minocycline,^[13]^ but whether drug induced lymphadenopathy should be considered as part of the same spectrum of disease as DIHS is under debate,^[14]^ since their exact pathophysiological mechanisms are not well understood and no common underlying mechanism has yet been confirmed. In this case, the histopathological findings along with those of PET scan and the clinical course of the patient after drug cessation prompted us to consider the possibility that drug-induced lymphadenopathy may be implicated in DAL.

The presence of PTGCs in our case, the etiology and pathogenesis of which remain unclear; along with immunoblastic proliferation may pose challenge to differential diagnosis mainly from Hodgkin lymphoma. PTGCs and nodular lymphocyte-predominant Hodgkin lymphoma share many histological features, with both entities presumed to be manifestations of an abnormal follicular center reaction and often correlated to each other. The presence of typical LP cells confirms the diagnosis of the lymphoma.^[15]^ However, in cases of viral- or drug- related PTGCs large cells may resemble LP or HRS cells making the differential diagnosis a challenge.^[15]^ In our case, immunoreactivity to T cell markers by few of the large cells, supported the possibility of immunoblastic reaction. In addition, there was no immunoreactivity to EMA antibody and the architecture and the surrounding to large cells microenvironment pointed to the correct diagnosis.^[15]^ Moreover, the coexistence of eosinophils and paracortical expansion in relation to clinical history favored the diagnosis of drug-induced lymphadenopathy. The resolution of lymphadenopathy following dasatinib discontinuation confirmed the diagnostic reasoning.

## 4 Conclusion

DAL may present as large mass. Interpretation of hypermetabolic lymphadenopathy detected by ^18^F-FDG PET/CT in this context should be cautious. On histological grounds, it may manifest with a spectrum of lesions including follicular and/or paracortical hyperplasia as observed in lymphadenopathy induced by other drugs. Further study is required to elucidate the pathogenesis of DAL.

Written informed consent was obtained from the patient for publication of this case report.

## Author contributions

All authors made substantial contributions to the acquisition, analysis, and interpretation of data. All authors were involved in drafting and revising the manuscript and have approved the published version.
